# Ferrostatin-1 Attenuates CLP-Induced Colonic Injury: Associations with Ferroptosis-Related Alterations, Oxidative Stress, Inflammation, and Treatment Timing

**DOI:** 10.3390/cimb48090894

**Published:** 2026-09-01

**Authors:** Mehmet Tolga Kafadar, Eda Yıldızhan

**Affiliations:** 1 Department of General Surgery, Faculty of Medicine, Dicle University, 21280 Diyarbakır, Turkey; 2 Department of Histology and Embryology, Faculty of Medicine, Recep Tayyip Erdogan University, 53100 Rize, Turkey; eda.yildizhan@erdogan.edu.tr

**Keywords:** Ferrostatin-1, ferroptosis, cecal ligation and puncture, fecal peritonitis, colonic injury, ACSL4, GPX4, oxidative stress

## Abstract

Fecal peritonitis induces severe oxidative and inflammatory injury and may involve ferroptosis-related mechanisms. This study investigated the effects of Ferrostatin-1 (Fer-1) on cecal ligation and puncture (CLP)-induced colonic injury, with emphasis on oxidative stress, inflammation, ferroptosis-related alterations, and treatment timing. Thirty-five male Wistar rats were randomly assigned to five groups: Sham, CLP, CLP + dimethyl sulfoxide (DMSO), CLP + Fer-1 (30 min), and CLP + Fer-1 (2 h). Fer-1 (5 mg/kg, intraperitoneally) was administered after CLP. Biochemical and inflammatory parameters, colonic histopathology, and acyl-CoA synthetase long-chain family member 4 (ACSL4) and glutathione peroxidase 4 (GPX4) immunoreactivity were evaluated. CLP induced marked oxidative and inflammatory alterations, increased histopathological damage and ACSL4 immunoreactivity, and decreased GPX4 immunoreactivity (all overall *p* < 0.001, except total antioxidant status, *p* = 0.004). Fer-1 treatment generally shifted these alterations toward the sham profile. In exploratory comparisons adjusted for multiple testing, the 2 h group showed lower malondialdehyde, tumor necrosis factor-alpha, and interleukin-6 levels and higher catalase concentrations than the 30 min group (Holm-adjusted *p* < 0.05). Group-adjusted analyses indicated that the strong pooled correlations were largely attributable to between-group differences. In silico analyses provided complementary hypothesis-generating molecular context. Fer-1 attenuated CLP-induced colonic injury and was associated with favorable oxidative–inflammatory changes and ferroptosis-related ACSL4/GPX4 alterations. However, the timing findings do not establish an optimal therapeutic window, and direct measures of ferroptotic cell death are required to clarify the contribution of ferroptosis-associated mechanisms.

## 1. Introduction

Colonic perforation is a life-threatening acute abdominal condition characterized by disruption of the colonic wall and leakage of fecal contents into the peritoneal cavity [1]. It is associated with high morbidity and mortality and most commonly occurs secondary to diverticulitis, malignancy, ischemic colitis, inflammatory bowel disease, and abdominal trauma [2,3]. The resulting polymicrobial peritonitis can rapidly progress to systemic inflammatory response syndrome (SIRS), sepsis, septic shock, and ultimately multiple organ dysfunction syndrome (MODS) [4].

Following fecal contamination, pathogen-associated molecular patterns (PAMPs) and damage-associated molecular patterns (DAMPs) activate innate immune responses and promote the release of pro-inflammatory cytokines [5,6], including tumor necrosis factor-alpha (TNF-α), interleukin-1 beta (IL-1β), and IL-6. This inflammatory response contributes to endothelial dysfunction, increased vascular permeability, microcirculatory impairment, and tissue injury [7]. Concurrently, excessive oxidative stress promotes membrane lipid peroxidation, compromising epithelial integrity and intestinal barrier function [8].

Ferroptosis is a distinct form of regulated cell death characterized by iron-dependent lipid peroxidation [9]. Two key molecular regulators of ferroptotic susceptibility are glutathione peroxidase 4 (GPX4) and acyl-CoA synthetase long-chain family member 4 (ACSL4) [10]. GPX4 protects cellular membranes by reducing lipid hydroperoxides, whereas ACSL4 promotes the incorporation of polyunsaturated fatty acids into membrane phospholipids, thereby increasing susceptibility to lipid peroxidation and ferroptosis [11,12]. Accordingly, reduced GPX4 activity and increased ACSL4 expression are closely associated with a ferroptosis-prone cellular state.

Accumulating evidence implicates ferroptosis in inflammatory disorders, sepsis, and gastrointestinal injury [13,14]. Oxidative stress and inflammation may also interact bidirectionally with ferroptotic processes, potentially amplifying tissue damage. Ferrostatin-1 (Fer-1), a radical-trapping antioxidant and widely used experimental ferroptosis inhibitor, suppresses the propagation of lipid peroxidation and has demonstrated protective effects in various models of oxidative and inflammatory injury [15,16]. However, its effects on colonic injury during fecal peritonitis, particularly when administered at different time points after the onset of injury, remain insufficiently characterized.

Therefore, this study aimed to investigate the effects of Fer-1 on CLP-induced colonic injury by evaluating oxidative stress, antioxidant defense, systemic inflammation, histopathological damage, and the ferroptosis-related markers ACSL4 and GPX4. Fer-1 was administered at two post-CLP time points to explore the potential influence of treatment timing. In addition, molecular docking, protein–protein interaction network, and functional enrichment analyses were performed to explore potential molecular interactions and pathways linking ferroptosis with inflammatory signaling.

## 2. Materials and Methods

### 2.1. Ethical Approval

All experimental procedures were approved by the Animal Experiments Local Ethics Committee of Dicle University (Approval No. 2026/05; Date: 26 March 2026, Türkiye). All procedures were conducted in accordance with the National Research Council’s Guide for the Care and Use of Laboratory Animals (8th edition) and complied with the ARRIVE 2.0 guidelines for reporting animal research [17]. Every effort was made to minimize animal suffering and to reduce the number of animals used throughout the study.

### 2.2. Animals, Housing Conditions, and Sample Size

The sample size was determined a priori using G*Power software (version 3.1, Heinrich Heine University Düsseldorf, Düsseldorf, Germany). A fixed-effects omnibus one-way ANOVA model with five experimental groups was used, assuming an effect size of f = 0.65, an α error probability of 0.05, and a statistical power (1 − β) of 0.80. The minimum required total sample size was calculated as 35 animals, corresponding to seven animals per group (actual power = 0.825). Thirty-five healthy adult male Wistar albino rats (8–10 weeks old) weighing approximately 250–300 g were obtained from the Experimental Animal Research Center of Dicle University. Animals were housed in polypropylene cages under standard laboratory conditions (22 ± 2 °C, 50–60% relative humidity, and a 12 h light/12 h dark cycle) with free access to standard pellet chow and tap water throughout the experimental period. Animals were allowed to acclimatize to the laboratory environment for one week before initiation of the experiments.

### 2.3. Experimental Design and Study Groups

Following the acclimatization period, animals were randomly allocated into five experimental groups (*n* = 7 per group) using a computer-generated randomization schedule.

Sham: Midline laparotomy was performed, and the cecum was gently exteriorized without ligation or puncture before abdominal closure.

CLP: Experimental polymicrobial peritonitis was induced using the standard cecal ligation and puncture (CLP) model.

CLP+DMSO: This group served as the vehicle control for the Fer-1 (30 min) treatment group. Experimental peritonitis and sepsis were induced using the CLP procedure, followed by a single intraperitoneal injection of 1% dimethyl sulfoxide (DMSO) 30 min after CLP. The vehicle was administered at the same injection volume as the Fer-1 preparation.

CLP + Fer-1 (30 min): Animals underwent CLP followed by intraperitoneal administration of Ferrostatin-1 (5 mg/kg) 30 min after surgery.

CLP + Fer-1 (2 h): Animals underwent CLP followed by intraperitoneal administration of Ferrostatin-1 (5 mg/kg) 2 h after surgery.

### 2.4. Blinding and Exclusion Criteria

Experimental procedures and outcome assessments were performed by different investigators. Group allocation was independently coded by an investigator who was not involved in outcome assessment, and investigators performing biochemical analyses, immunohistochemical quantification, and histopathological evaluation were blinded to group allocation during outcome assessment. Predefined exclusion criteria included perioperative mortality, anesthesia-related death, and major surgical complications that could compromise completion of the experimental protocol or subsequent outcome assessment. No animals died or met the predefined exclusion criteria after randomization; therefore, all 35 randomized animals completed the experimental protocol and were included in the final analyses.

### 2.5. Induction of Fecal Peritonitis by CLP

Experimental polymicrobial peritonitis was induced using the CLP model as previously described by Rittirsch et al. with minor modifications. Following an overnight fast with free access to water, rats were anesthetized by intraperitoneal administration of ketamine hydrochloride (90 mg/kg; Ketalar^®^, Pfizer Inc., New York, NY, USA; sourced commercially in Türkiye) and xylazine hydrochloride (10 mg/kg; Rompun^®^, Bayer HealthCare AG, Leverkusen, Germany). Adequate anesthesia was confirmed by the absence of the pedal withdrawal reflex. After adequate anesthesia had been achieved, the abdominal area was shaved and disinfected with povidone–iodine solution.

A 2–3 cm midline laparotomy was performed under sterile conditions. The cecum was carefully exteriorized while preserving the mesenteric blood supply and ligated distal to the ileocecal valve without causing intestinal obstruction. The ligated cecum was punctured once in a through-and-through manner using an 18-gauge needle, and a small amount of fecal material was gently extruded through the puncture site to ensure patency.

The cecum was then returned to the abdominal cavity without contaminating the abdominal wound margins, and the abdominal musculature and skin were closed separately using absorbable sutures. Postoperative analgesia was provided with a single subcutaneous dose of meloxicam (1–2 mg/kg). Animals also received a single subcutaneous administration of warmed 0.9% NaCl (3–5 mL/100 g body weight) for fluid resuscitation. Sham-operated animals underwent identical surgical procedures except for cecal ligation and puncture. All animals were euthanized 6 h after CLP induction for sample collection [14].

### 2.6. Ferrostatin-1 Preparation and Administration

Ferrostatin-1 (Fer-1; purity > 98%; Cat. No. BD206880-1g; BLDpharm, Shanghai, China) was obtained commercially and dissolved in 1% DMSO immediately before administration. Based on previous experimental studies investigating ferroptosis inhibition, Fer-1 was administered as a single intraperitoneal injection at a dose of 5 mg/kg [9]. Two post-CLP administration time points (30 min and 2 h) were selected to explore whether the timing of Fer-1 administration influenced its effects on CLP-induced injury [9,11]. These time points were evaluated as exploratory treatment schedules rather than as predefined therapeutic windows [7].

### 2.7. Sample Collection and Study Termination

Six hours after CLP induction, all animals were anesthetized with ketamine hydrochloride (90 mg/kg) and xylazine hydrochloride (10 mg/kg). Blood samples were collected by intracardiac puncture prior to euthanasia. Animals were subsequently euthanized by exsanguination under deep anesthesia. Colon samples were collected in a standardized manner from the proximal colonic segment immediately adjacent to the cecum and processed for subsequent histopathological and immunohistochemical analyses. Tissue samples designated for histological evaluation were fixed in 10% neutral-buffered formalin, whereas serum samples were separated by centrifugation and stored at −80 °C until biochemical analysis.

### 2.8. Biochemical Analyses

Blood samples collected into serum separation tubes were allowed to clot for 10 min at room temperature. Samples were centrifuged at 3000 rpm for 15 min using a refrigerated centrifuge (Rotanta 460, Hettich, Tuttlingen, Germany), and the sera were separated and stored at −80 °C until analysis. Serum C-reactive protein (CRP) levels were determined according to the manufacturer’s instructions, and the results were expressed as mg/L [18]. Serum lactate dehydrogenase (LDH) activity was measured using an automated biochemical analyzer according to the manufacturer’s protocol and expressed as U/L [14]. Serum total antioxidant status (TAS) and total oxidant status (TOS) were determined spectrophotometrically using commercially available assay kits (Rel Assay Diagnostics, Gaziantep, Türkiye) according to the methods developed by Erel [19,20]. Analyses were performed using an AU5800 automated biochemical analyzer (Beckman Coulter Inc., Brea, CA, USA). TAS values were expressed as mmol Trolox equivalent/L, whereas TOS values were expressed as μmol H_2_O_2_ equivalent/L. The oxidative stress index (OSI) was calculated using the following formula: OSI = [TOS (μmol H_2_O_2_ Eq/L)/TAS (mmol Trolox Eq/L)] × 100. OSI values were expressed as arbitrary units (AU) (16).

Serum malondialdehyde (MDA), glutathione (GSH), superoxide dismutase 1 (SOD1), and catalase (CAT) levels were determined using rat-specific enzyme-linked immunosorbent assay (ELISA) kits (Bioassay Technology Laboratory, Jiaxing, China) according to the manufacturer’s instructions. All ELISA washing procedures were performed using a BioTek ELx50 Microplate Washer (Instruments Inc., Winooski, VT, USA), and absorbance was measured with a BioTek ELx800 Microplate Reader (BioTek Instruments Inc., Winooski, VT, USA). Concentrations were calculated from standard calibration curves according to the manufacturers’ protocols.

Serum TNF-α and IL-6 concentrations were quantified using rat-specific ELISA kits (Bioassay Technology Laboratory, Jiaxing, China) according to the manufacturer’s instructions. Absorbance values were measured using a BioTek ELx800 Microplate Reader, and cytokine concentrations were calculated from standard calibration curves provided by the manufacturer. Details of the biochemical parameters, analytical methods, assay kits, manufacturers, catalog numbers, and measurement units are provided in Supplementary Table S1.

### 2.9. Histological Examination

Colon tissue samples were fixed in 10% neutral-buffered formalin for 24–48 h, routinely processed through graded ethanol and xylene, embedded in paraffin, and sectioned at a thickness of 4–5 μm using a rotary microtome. Tissue sections were stained with hematoxylin and eosin (H&E) according to standard histological procedures. Histopathological evaluation was performed under a light microscope (Olympus BX53, Olympus Corporation, Tokyo, Japan) by an experienced histologist blinded to the experimental groups. Colonic injury was assessed by examining mucosal architecture, epithelial integrity, crypt morphology, inflammatory cell infiltration, submucosal edema, vascular congestion, hemorrhage, and necrosis. The severity of intestinal injury was evaluated using the semi-quantitative histopathological scoring system described by Chiu et al. [21]. This grading system classifies mucosal injury from Grade 0 (normal mucosal architecture) to Grade 5 (complete destruction of villous architecture with extensive ulceration and disintegration of the lamina propria). Representative images were captured using a digital microscope imaging system under identical acquisition settings for all groups.

### 2.10. Immunohistochemical Examination

Immunohistochemical staining was performed to evaluate the tissue expression of (GPX4 and ACSL4. Paraffin-embedded colon tissue sections (4–5 μm) were deparaffinized in xylene and rehydrated through graded ethanol solutions. Antigen retrieval was performed using citrate buffer (pH 6.0) in a microwave oven. Endogenous peroxidase activity was blocked with 3% hydrogen peroxide, followed by incubation with a protein blocking solution to minimize nonspecific binding. Tissue sections were incubated overnight at 4 °C with primary antibodies against GPX4 (sc-166570, Santa Cruz Biotechnology, Dallas, TX, USA) and ACSL4 (sc-365230, Santa Cruz Biotechnology, Dallas, TX, USA). After washing with phosphate-buffered saline (PBS), sections were incubated with an appropriate horseradish peroxidase-conjugated secondary antibody. Immunoreactivity was visualized using 3,3′-diaminobenzidine (DAB) as the chromogen, and nuclei were counterstained with Mayer’s hematoxylin. Immunostained sections were examined under identical microscopic conditions by an investigator blinded to the experimental groups. Digital images were acquired using identical illumination and exposure settings. Immunoreactivity was quantified using the H-score method. Five randomly selected non-overlapping high-power fields (×400 magnification) were analyzed for each tissue section. Staining intensity was classified as weak (1+), moderate (2+), or strong (3+). The H-score was calculated as (1× percentage of weakly stained cells) + (2× percentage of moderately stained cells) + (3× percentage of strongly stained cells), yielding a theoretical score ranging from 0 to 300 [22].

### 2.11. In Silico Analyses

#### 2.11.1. Molecular Docking Analysis

Molecular docking was performed to evaluate the potential interactions of Fer-1 with ACSL4, GPX4, TNF-α, and IL-6. Protein structures were obtained from the RCSB Protein Data Bank, including TNF-α (PDB ID: 1TNF), GPX4 (PDB ID: 6HN3) and IL-6 (PDB ID: 1ALU), while the full-length ACSL4 structure was obtained from AlphaFold due to the absence of an experimentally resolved full-length structure. The Fer-1 structure was retrieved from PubChem. Receptors and the ligand were prepared by removing water molecules and non-essential heteroatoms, adding polar hydrogens and Gasteiger charges, and converting the structures to PDBQT format. For GPX4, Sec46 was modeled as cysteine to ensure compatibility with the docking workflow. Docking was performed using AutoDock Vina v1.2 with protein-specific search spaces encompassing the respective binding regions, an exhaustiveness value of 32, an energy range of 4 kcal/mol, and 20 generated poses. The lowest-energy pose for each target was selected for analysis. The highest-ranked Fer-1–ACSL4 complex was visualized using PyMOL (version 3.2.0; Schrödinger, LLC, New York, NY, USA), and residues within 4 Å of the ligand were identified to characterize the predicted binding pocket [23,24]. The predicted Fer-1–ACSL4 docking pose and residues located within 4 Å of the ligand are presented in Supplementary Figure S1.

#### 2.11.2. Protein–Protein Interaction (PPI) Network Analysis

A protein–protein interaction (PPI) network was constructed using the STRING database (version 12.0; https://string-db.org, accessed on 11 July 2026) to characterize the interaction landscape of the predefined ferroptosis- and inflammation-related proteins. A total of 23 proteins (ACSL4, GPX4, SLC7A11, SLC3A2, LPCAT3, ALOX15, TFRC, NCOA4, FTH1, FTL, GCLC, GCLM, NFE2L2, KEAP1, HMOX1, PTGS2, TLR4, MYD88, NFKB1, RELA, TNF, IL1B, and IL6) were entered as input. Homo sapiens was selected as the reference organism, the minimum required interaction score was set to high confidence (0.700), and no additional interactors were added. The resulting network was imported into Cytoscape (version 3.10.4; Cytoscape Consortium, USA) for visualization and network analysis. Highly interconnected modules were identified using the Molecular Complex Detection (MCODE) plugin with default parameters (degree cutoff = 2, node score cutoff = 0.2, k-core = 2, and maximum depth = 100). Functional enrichment analysis was performed for KEGG pathways and Gene Ontology Cellular Component (GO-CC) terms [25,26].

#### 2.11.3. Functional Enrichment Analysis

Functional enrichment analysis of the predefined protein set was performed to provide functional context for the PPI network. Kyoto Encyclopedia of Genes and Genomes (KEGG) pathway enrichment and Gene Ontology Cellular Component (GO-CC) enrichment analyses were evaluated. Significantly enriched terms were identified using multiple-testing-adjusted *p* values, with an adjusted *p* value < 0.05 considered statistically significant. The enrichment results were interpreted as descriptive and hypothesis-generating because the input proteins had been predefined based on their known involvement in ferroptosis, oxidative stress, and inflammatory signaling [27]. Functional enrichment analysis of the predefined protein set identified significant enrichment of pathways associated with innate immune activation and inflammatory signaling (Supplementary Figure S2).

### 2.12. Statistical Analysis

Statistical analyses were performed using IBM SPSS Statistics for Windows, Version 27.0 (IBM Corp., Armonk, NY, USA). The distribution of continuous variables was assessed for normality with Shapiro–Wilk test, and because the data did not meet the assumptions required for parametric testing, continuous variables were presented as median (minimum–maximum). A single primary outcome was not prespecified in the experimental protocol. Accordingly, the biochemical, inflammatory, histopathological, and immunohistochemical outcomes were evaluated within an exploratory analytical framework. Direct comparisons between the two Fer-1 treatment schedules and correlation analyses were also considered exploratory.

Differences among the five experimental groups were analyzed using the Kruskal–Wallis test, followed by Dunn’s post hoc test with Bonferroni adjustment for multiple pairwise comparisons. Effect sizes for the Kruskal–Wallis analyses were expressed as epsilon squared (ε^2^). Associations among biochemical, oxidative stress, inflammatory, immunohistochemical, and histopathological parameters were evaluated using Spearman’s rank correlation analysis and reported as Spearman’s correlation coefficients (r_s_).

Initial associations among biochemical, oxidative stress, inflammatory, immunohistochemical, and histopathological parameters were explored using Spearman’s rank correlation analysis. Because pooled correlations across experimentally distinct groups could be influenced by between-group differences, a sensitivity analysis was performed using partial correlations adjusted for experimental group membership. Group membership was represented by four dummy variables, with the Sham group as the reference category. The group-adjusted analyses were used for the primary interpretation of the correlation findings. In addition, the two Fer-1 treatment groups were directly compared using the Mann–Whitney U test as an exploratory analysis of treatment timing. To account for multiple testing across the 14 treatment-timing comparisons, *p* values were adjusted using the Holm procedure. Effect sizes for these comparisons were calculated as r = |Z|/√N. All statistical tests were two-tailed, and *p* < 0.05 was considered statistically significant.

## 3. Results

### 3.1. Biochemical and Oxidative Stress Findings

Significant differences were observed among the experimental groups for all evaluated biochemical and oxidative-stress parameters (Table 1). CRP, LDH, TOS, OSI, and MDA levels were markedly elevated in the CLP and CLP+DMSO groups compared with the sham group, whereas antioxidant parameters, including GSH, SOD1, and CAT, were reduced (all overall *p* < 0.001). TAS also differed significantly among the groups (*p* = 0.004). The CLP and CLP+DMSO groups generally exhibited comparable biochemical profiles, indicating no detectable protective effect of the CLP + vehicle.

Fer-1 treatment was associated with a shift toward the sham profile, although the magnitude of the differences varied according to the parameter and treatment timing. In the Dunn–Bonferroni analysis, the Fer-1 (2 h) group had significantly lower CRP and OSI levels than the CLP group and significantly lower LDH levels than both the CLP and CLP+DMSO groups. The Fer-1 (2 h) group also showed significantly higher TAS than the CLP group and higher GSH, SOD1, and CAT levels than the CLP+DMSO group; CAT was additionally higher than in the CLP group. Although Fer-1 treatment produced directional improvements in several other oxidative-stress parameters, some pairwise comparisons did not remain significant after Bonferroni adjustment. Effect sizes for the overall group differences were large, with ε^2^ values ranging from 0.452 to 0.827.

### 3.2. Inflammatory Cytokine Findings

Serum TNF-α and IL-6 levels differed significantly among the experimental groups (both *p* < 0.001), with large effect sizes (ε^2^ = 0.821 and 0.874, respectively; Table 2). Both cytokines were markedly elevated in the CLP and CLP+DMSO groups compared with the sham group. TNF-α levels were significantly lower in the Fer-1 (2 h) group than in both the CLP and CLP+DMSO groups. Similarly, IL-6 was significantly lower in the Fer-1 (2 h) group than in the CLP group. The Fer-1 (30 min) group showed intermediate cytokine levels; however, its differences from the CLP and CLP+DMSO groups did not reach statistical significance after Bonferroni adjustment.

### 3.3. Histopathological Analysis

**Figure 1:**
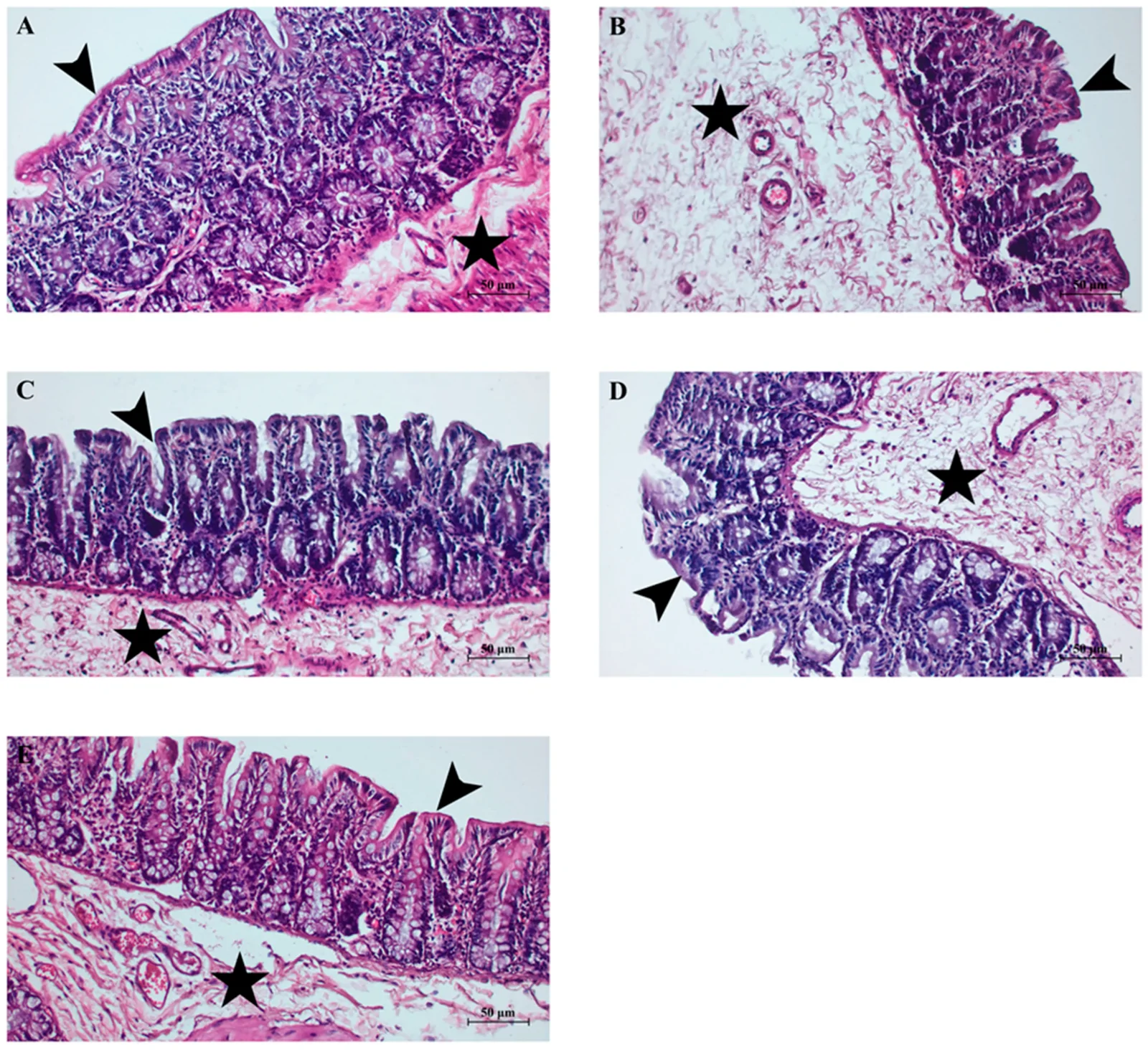
Representative H&E-stained sections of colon tissue from the experimental groups. (**A**) Sham group showing preserved epithelial integrity (arrowhead) and normal connective tissue architecture (star). (**B**) CLP group demonstrating disruption of the epithelial lining and marked loosening of the connective tissue. (**C**) CLP + DMSO group showing persistent histopathological alterations similar to those observed in the CLP group. (**D**) CLP + Fer-1 (30 min) group showing preservation of mucosal architecture and reduced connective tissue injury. (**E**) CLP + Fer-1 (2 h) group demonstrating improved epithelial integrity and connective tissue organization compared with the untreated CLP group. Arrowheads indicate the epithelial lining, and stars indicate the underlying connective tissue. H&E staining; scale bar = 50 μm.

Representative H&E-stained colon sections are presented in Figure 1. The sham group demonstrated well-preserved colonic histology. The epithelial lining (arrowhead) was continuous and intact with normal crypt organization. The underlying connective tissue (star) exhibited normal compact architecture without evidence of edema or structural disorganization (Figure 1A). The CLP group exhibited pronounced histopathological injury. The epithelial lining appeared irregular with distortion of the mucosal architecture. The connective tissue showed marked loosening with a pale appearance compatible with edema and loss of normal stromal organization. Overall tissue integrity was markedly compromised compared with the sham group (Figure 1B). Histological findings in the DMSO-treated group were largely comparable to those observed in the CLP group. Although the epithelial lining remained partially continuous, structural abnormalities persisted, and the underlying connective tissue exhibited loosening and mild edema, indicating that CLP+DMSO administration did not substantially attenuate CLP-induced tissue injury (Figure 1C). Administration of Fer-1 (30 min) after CLP resulted in marked preservation of colonic architecture. Although mild epithelial irregularity remained, the mucosal structure was substantially better preserved than in the CLP group. The connective tissue demonstrated reduced loosening and improved structural organization, indicating attenuation of CLP-induced histopathological damage (Figure 1D). The group receiving Fer-1 (2 h) also demonstrated considerable histological improvement compared with the untreated CLP group. The epithelial lining was largely preserved with only mild irregularity, and the connective tissue appeared more organized with less evidence of edema. Although slight architectural alterations remained, overall tissue morphology was closer to that observed in the sham group than in the CLP and DMSO groups (Figure 1E).

### 3.4. Immunohistochemical Analysis

**Figure 2:**
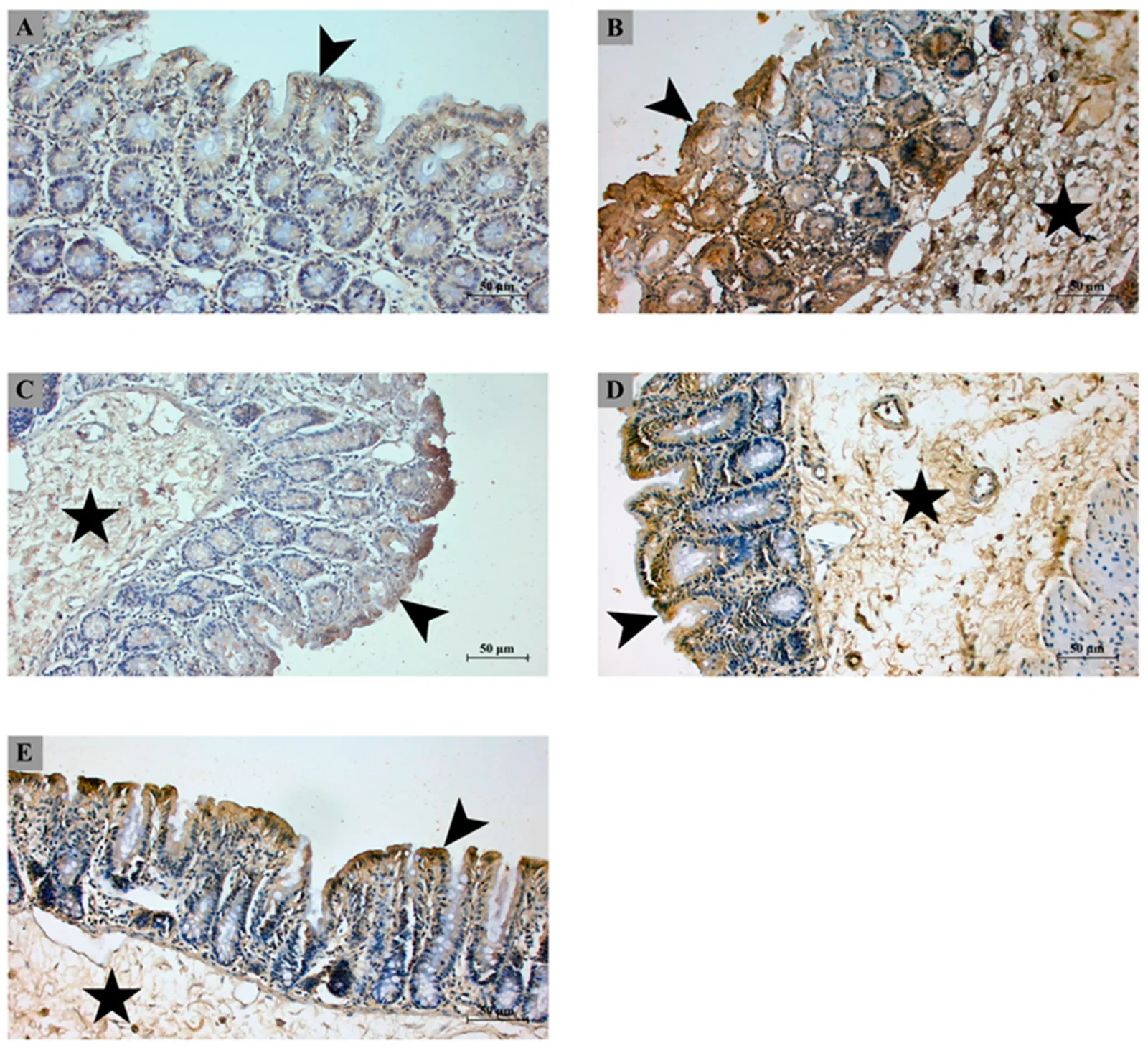
Representative immunohistochemical staining of ACSL4 in colon tissues from the experimental groups. (**A**) Sham group showing weak epithelial cytoplasmic immunoreactivity. (**B**) CLP group demonstrating markedly increased ACSL4 immunoreactivity in the epithelial lining. (**C**) CLP+DMSO group exhibiting persistent ACSL4 immunoreactivity comparable to the CLP group. (**D**) CLP + Fer-1 (30 min) group showing reduced epithelial ACSL4 immunoreactivity. (**E**) CLP + Fer-1 (2 h) group demonstrating attenuated ACSL4 immunoreactivity compared with the untreated CLP group. Arrowheads indicate the epithelial lining, and stars indicate the underlying connective tissue. Immunohistochemistry; scale bar = 50 μm.

Representative ACSL4-immunostained colon sections are presented in Figure 2. The sham group exhibited weak ACSL4 immunoreactivity. Faint cytoplasmic staining was observed in the epithelial cells lining the mucosa (arrowhead), while the underlying connective tissue (star) was largely immunonegative. The overall staining pattern was sparse and uniformly distributed (Figure 2A). The CLP group demonstrated a marked increase in ACSL4 immunoreactivity. Moderate-to-strong brown cytoplasmic staining was evident throughout the epithelial lining, with diffuse positivity extending along the crypt epithelium. The connective tissue remained largely negative, although the surrounding stromal architecture appeared loosened compared with the sham group (Figure 2B). The DMSO-treated group exhibited an immunostaining pattern comparable to that of the CLP group. Moderate cytoplasmic ACSL4 positivity persisted within the epithelial compartment, with no obvious reduction in staining intensity following CLP+DMSO administration (Figure 2C). Administration of Fer-1 (30 min) after CLP noticeably reduced ACSL4 immunoreactivity. The epithelial lining exhibited predominantly weak-to-moderate cytoplasmic staining, and the overall distribution of positive cells was less extensive than that observed in the CLP and DMSO groups. The connective tissue remained largely immunonegative (Figure 2D). The Fer-1 (2 h) treatment group also demonstrated reduced ACSL4 immunoreactivity compared with the untreated CLP group. Mild-to-moderate epithelial cytoplasmic immunoreactivity was observed, with an overall staining pattern consistent with the reduced ACSL4 H-score observed in this group. Tissue architecture was better preserved than in the CLP and DMSO groups (Figure 2E).

**Figure 3:**
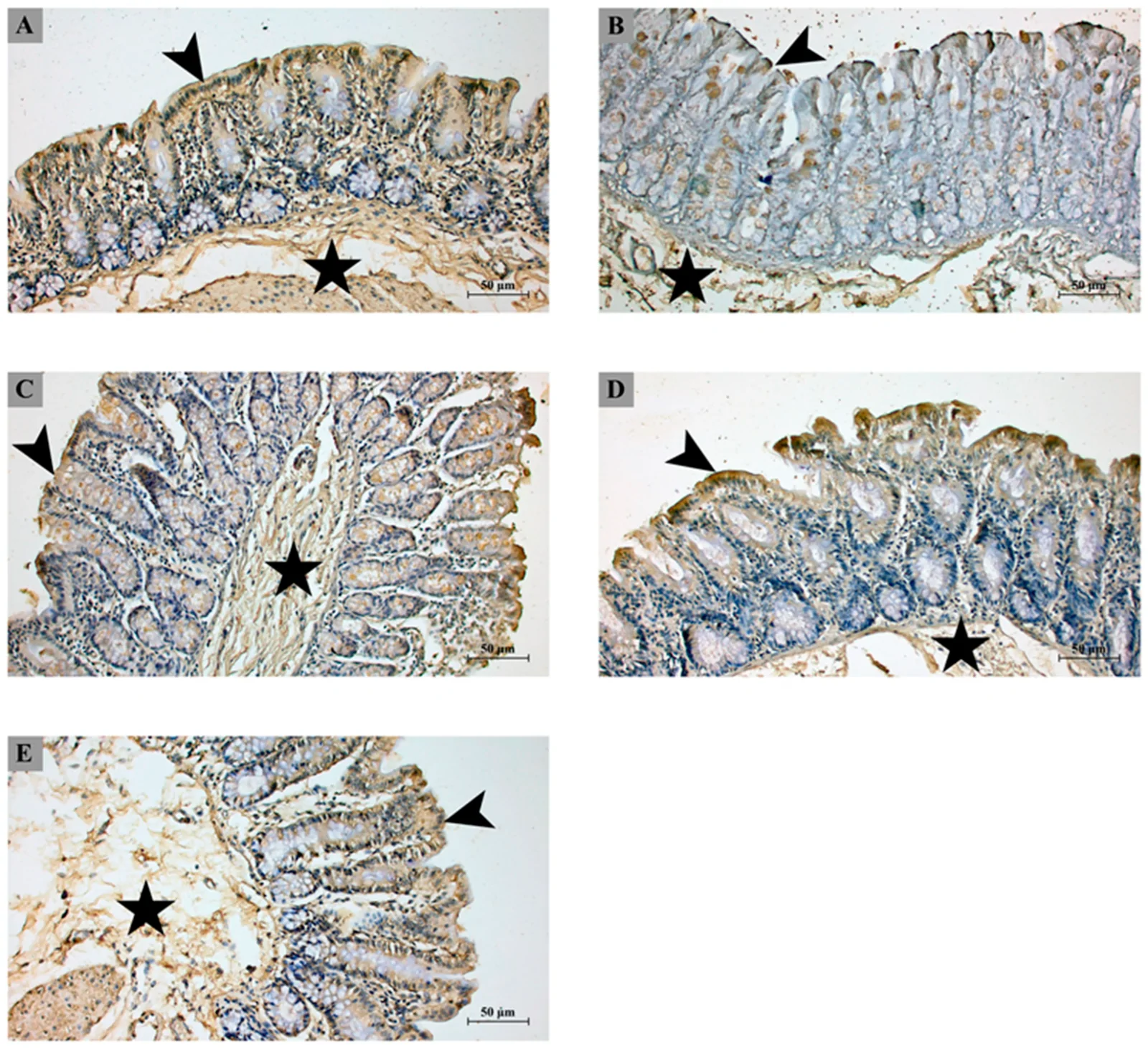
Representative immunohistochemical staining of GPX4 in colon tissues from the experimental groups. (**A**) Sham group showing strong epithelial cytoplasmic GPX4 immunoreactivity. (**B**) CLP group demonstrating markedly reduced GPX4 immunoreactivity. (**C**) CLP+DMSO group showing persistently weak GPX4 immunoreactivity comparable to the CLP group. (**D**) CLP + Fer-1 (30 min) group exhibiting increased epithelial GPX4 immunoreactivity. (**E**) CLP + Fer-1 (2 h) group demonstrating increased GPX4 immunoreactivity compared with the untreated CLP group. Arrowheads indicate the epithelial lining, and stars indicate the underlying connective tissue. Immunohistochemistry; scale bar = 50 μm.

Representative GPX4-immunostained colon sections are shown in Figure 3. The sham group exhibited strong and diffuse cytoplasmic GPX4 immunoreactivity throughout the colonic epithelial lining (arrowhead). Positive staining was predominantly localized within the epithelial cells, whereas the underlying connective tissue (star) showed minimal immunoreactivity. The staining pattern was homogeneous and continuous along the mucosal surface (Figure 3A). The CLP group demonstrated a marked reduction in GPX4 immunoreactivity. Only weak cytoplasmic staining was observed in scattered epithelial cells, and the overall staining intensity was considerably lower than that of the sham group. The connective tissue remained largely immunonegative (Figure 3B). The DMSO-treated group exhibited a staining pattern similar to that observed in the CLP group. GPX4 immunoreactivity remained weak within the epithelial lining, indicating that CLP+DMSO administration did not restore GPX4 expression following CLP-induced injury (Figure 3C). Treatment with Fer-1 at 30 min after CLP markedly increased GPX4 immunoreactivity. Moderate-to-strong cytoplasmic staining was observed throughout the epithelial lining, with a more continuous distribution of positively stained epithelial cells compared with the CLP and DMSO groups. The staining pattern approached that observed in the sham group (Figure 3D). Administration of Fer-1 at 2 h also enhanced GPX4 immunoreactivity compared with the untreated CLP group. Moderate-to-strong cytoplasmic immunoreactivity was observed within the epithelial cells, consistent with the higher GPX4 H-score observed in this group relative to the untreated CLP group. GPX4 immunoreactivity remained clearly greater than that observed in the CLP and DMSO groups (Figure 3E).

### 3.5. Histopathological and Immunohistochemical Findings

Significant group differences were also observed in histopathological scores and ACSL4 and GPX4 H-scores (all *p* < 0.001), with large effect sizes (ε^2^ = 0.848, 0.861, and 0.852, respectively; Table 3). Histopathological damage and ACSL4 immunoreactivity were markedly increased, whereas GPX4 immunoreactivity was reduced, in the CLP and CLP+DMSO groups compared with the Sham group.

The Fer-1 (2 h) group showed significantly lower histopathological scores than both the CLP and CLP+DMSO groups. ACSL4 H-scores were significantly lower in the Fer-1 (2 h) group than in the CLP group, whereas GPX4 H-scores were significantly higher. Although the Fer-1 (30 min) group showed numerically lower histopathological damage and ACSL4 immunoreactivity and higher GPX4 immunoreactivity than the untreated CLP group, these pairwise differences did not remain significant after Bonferroni correction. Overall, CLP-induced tissue injury was characterized by increased ACSL4 and reduced GPX4 immunoreactivity, whereas Fer-1 treatment was associated with attenuation of these alterations.

### 3.6. Group-Adjusted Correlation Analysis

Because correlations across all animals could be influenced by experimentally induced between-group differences, a sensitivity analysis was performed using partial correlations adjusted for experimental group membership. After adjustment, the strong associations observed in the initial pooled Spearman analysis were substantially attenuated (Table 4). ACSL4 H-score was not significantly associated with CRP, LDH, TAS, TOS, OSI, MDA, GSH, SOD1, CAT, GPX4 H-score, or histopathological score after adjustment for experimental group (all *p* > 0.05). Nominal inverse associations were observed between ACSL4 H-score and TNF-α (r = −0.357, *p* = 0.049) and IL-6 (r = −0.382, *p* = 0.034). GPX4 H-score and histopathological score showed no significant group-adjusted associations with the evaluated biochemical or inflammatory parameters (all *p* > 0.05). Overall, the marked attenuation of the pooled correlations after adjustment indicates that the original associations were predominantly driven by between-group experimental differences rather than independent within-group relationships.

### 3.7. Comparison Between Fer-1 Administration Times

Direct comparisons between the Fer-1 (30 min) and Fer-1 (2 h) groups were considered exploratory and were adjusted for multiple testing using the Holm procedure across 14 outcomes (Table 5). Before adjustment, differences were observed for several biochemical, inflammatory, immunohistochemical, and histopathological parameters. After Holm correction, four outcomes remained statistically significant. Compared with the Fer-1 (30 min) group, the Fer-1 (2 h) group showed lower MDA levels (Holm-adjusted *p* = 0.044), higher CAT concentrations (adjusted *p* = 0.028), and lower TNF-α and IL-6 levels (both adjusted *p* = 0.039). Differences in CRP, LDH, GSH, SOD1, GPX4 H-score, and histopathological score did not remain significant after multiplicity adjustment (all adjusted *p* > 0.05). TAS, TOS, OSI, and ACSL4 H-score also did not differ significantly between the two treatment groups. These findings indicate selected oxidative and inflammatory differences between the two administration schedules but do not establish superiority of either treatment time.

### 3.8. Molecular Analysis

Molecular docking analysis was performed to explore potential interactions between Fer-1 and selected ferroptosis- and inflammation-related target proteins. Among the evaluated proteins, Fer-1 yielded the most favorable predicted docking score for ACSL4 (−6.816 kcal/mol), followed by IL-6 (−5.541 kcal/mol), GPX4 (−5.322 kcal/mol), and TNF-α (−4.251 kcal/mol). Thus, ACSL4 showed the most favorable computationally predicted interaction among the four selected targets, whereas TNF-α showed the least favorable docking score. These findings represent in silico predictions and should not be interpreted as evidence of direct molecular binding or target engagement. Overall, the docking analysis identified differences in predicted interaction scores among the selected targets and provides a hypothesis-generating basis for further experimental investigation.

### 3.9. Protein–Protein Interaction Network Analysis

**Figure 4:**
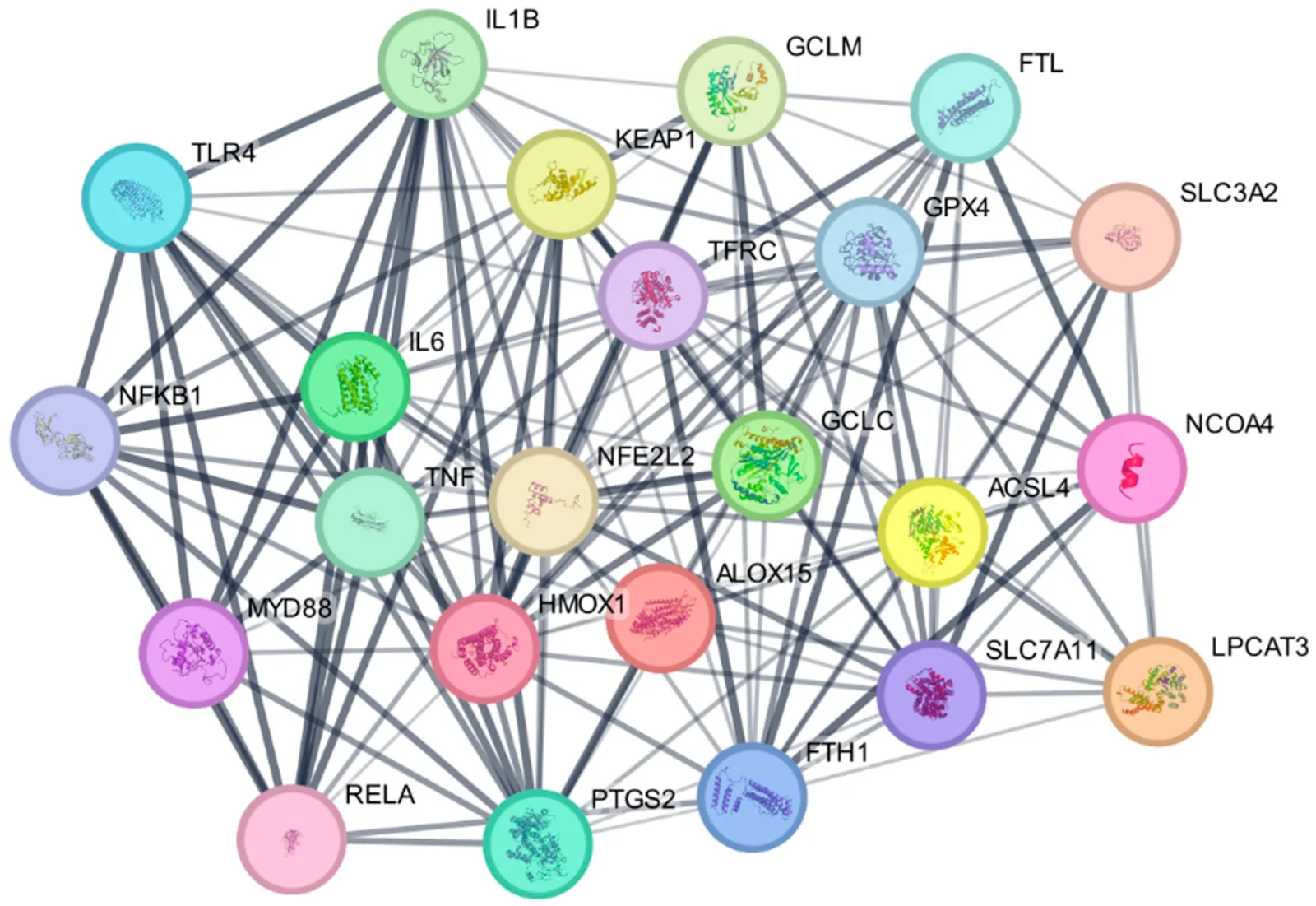
PPI network of the predefined ferroptosis- and inflammation-related proteins. Highly interconnected proteins formed a core module comprising proteins associated with ferroptosis-related processes, oxidative-stress regulation, and inflammatory signaling.

The predefined protein set formed an interconnected PPI network, within which MCODE identified two highly connected modules. The highest-scoring module had an MCODE score of 8.909 and contained 12 proteins: ACSL4, NCOA4, NFE2L2, KEAP1, PTGS2, TLR4, MYD88, NFKB1, RELA, TNF, IL1B, and IL6 (Figure 4). These proteins represented a network context linking ferroptosis-related, oxidative-stress, and inflammatory signaling components. Because the network was generated from an a priori selected protein set, these findings were considered functional contextualization of the selected targets rather than independent evidence of a common regulatory mechanism.

## 4. Discussion

Fecal peritonitis is characterized by polymicrobial contamination, systemic inflammation, oxidative stress, and progressive tissue injury [28]. In the present study, CLP induced a pronounced oxidative–inflammatory response accompanied by colonic damage, increased ACSL4 immunoreactivity, and decreased GPX4 immunoreactivity. Fer-1 treatment shifted several of these alterations toward the sham profile, while exploratory comparison of the two administration schedules identified differences in selected oxidative and inflammatory outcomes. These findings support the involvement of ferroptosis-related mechanisms in CLP-induced colonic injury. In a directly comparable study, Hou et al. [29] demonstrated that Fer-1 administered at 5 mg/kg in CLP-induced sepsis reduced intestinal morphological injury, systemic inflammation, oxidative stress, and ferroptosis-related alterations while improving intestinal barrier-related markers. Similarly, Ma et al. [30] demonstrated the involvement of Nrf2/GPX4-mediated ferroptosis in CLP-induced intestinal injury and showed that modulation of ferroptosis influenced both inflammatory responses and intestinal barrier function. Together, these observations support ferroptosis-associated lipid injury as a potential contributor to intestinal damage during polymicrobial sepsis. However, the treatment-timing findings should be interpreted cautiously. After Holm correction for multiple comparisons, only MDA, CAT, TNF-α, and IL-6 remained significantly different between the two Fer-1 groups. Moreover, because all animals were evaluated at the fixed 6 h endpoint, the treatment-to-sampling interval differed between the 30 min and 2 h groups (approximately 5.5 and 4 h, respectively). Thus, these differences may partly reflect pharmacokinetic or pharmacodynamic proximity to sampling rather than a true advantage of delayed administration. Given that only two treatment time points were examined, these findings should be considered exploratory and hypothesis-generating rather than evidence of an optimal therapeutic window.

CLP caused marked increases in CRP, LDH, TOS, OSI, MDA, TNF-α, and IL-6, together with depletion of antioxidant defenses, including GSH, SOD1, and CAT. Fer-1 generally shifted this oxidative–inflammatory profile toward that of the sham group, although not all pairwise comparisons remained significant after adjustment for multiple testing. Fer-1 acts primarily as a radical-trapping antioxidant that interrupts phospholipid peroxidation chain reactions, as established by Dixon et al. [9] and further characterized by Skouta et al. [31] Accordingly, the lower MDA levels and preservation of antioxidant defenses observed following Fer-1 treatment are consistent with attenuation of lipid peroxidation-mediated injury. The accompanying reductions in TNF-α and IL-6 also support an interaction between ferroptotic lipid damage and inflammation. In polymicrobial sepsis, Kang et al. [32] demonstrated that GPX4 restrains lipid peroxidation-dependent inflammatory cell death and cytokine release, providing a mechanistic basis for the parallel changes in lipid peroxidation and inflammatory markers observed in the present study.

The histopathological and immunohistochemical findings were consistent with the biochemical profile. CLP caused substantial disruption of colonic architecture, whereas Fer-1 treatment was associated with improved epithelial integrity and tissue organization. These findings parallel those of Hou et al. [29], who demonstrated that Fer-1 attenuated intestinal morphological damage and preserved tight-junction proteins in CLP-induced sepsis. Furthermore, Liu et al. [33] reported that suppression of ferroptosis alleviated septic intestinal inflammation and barrier dysfunction, supporting a broader relationship between ferroptotic injury and intestinal epithelial integrity. At the molecular level, the increased ACSL4 and decreased GPX4 immunoreactivity observed following CLP are consistent with a ferroptosis-permissive phenotype. Doll et al. [34] established ACSL4 as a major determinant of ferroptosis sensitivity through its role in shaping PUFA-containing membrane phospholipids, while Yang et al. [35] identified GPX4 as a central suppressor of ferroptotic lipid peroxidation. More specifically, Li et al. [36] demonstrated that ACSL4 activation contributes directly to ferroptosis-mediated intestinal tissue injury, while Ma et al. [30] implicated the Nrf2/GPX4 pathway in sepsis-associated intestinal damage. Thus, the reciprocal ACSL4 and GPX4 alterations observed in the present study provide tissue-level support for ferroptosis-related involvement, although direct evidence of ferroptotic cell death would require complementary measurements of iron accumulation, lipid ROS, and ultrastructural alterations. Although the reciprocal alterations in ACSL4 and GPX4 immunoreactivity, together with the oxidative-stress profile, are consistent with involvement of ferroptosis-associated pathways, these findings should not be interpreted as direct evidence of ferroptotic cell death. Fer-1 is also a radical-trapping antioxidant, and the present experimental design does not allow its protective effects to be attributed exclusively to ferroptosis inhibition.

The initial pooled correlation analysis showed strong associations among ACSL4 and GPX4 immunoreactivity, oxidative–inflammatory markers, and histopathological damage. However, these associations were substantially attenuated after adjustment for experimental group membership. In the group-adjusted analysis, ACSL4 H-score showed only nominal inverse associations with TNF-α and IL-6, whereas the remaining associations involving ACSL4, GPX4, and histopathological scores were not statistically significant. This attenuation indicates that the strong pooled correlations were largely attributable to experimentally induced between-group differences rather than independent within-group relationships. Accordingly, the correlation findings should be interpreted as reflecting coordinated group-level responses to CLP and Fer-1 treatment rather than evidence of direct biological coupling among these markers.

The in silico analyses provided complementary mechanistic hypotheses. Among the investigated targets, Fer-1 yielded the most favorable predicted docking score for ACSL4. However, existing experimental studies, including those of Dixon et al. [9] and Skouta et al. [31], primarily characterize Fer-1 as a radical-trapping inhibitor of oxidative lipid damage, and direct binding to or enzymatic inhibition of ACSL4 has not been established. The predicted Fer-1 docking pose with ACSL4 should therefore be regarded as a hypothesis-generating observation requiring experimental target-validation studies. The PPI and enrichment analyses additionally highlighted NF-κB, Toll-like receptor, NOD-like receptor, and IL-17 signaling. Supporting the biological plausibility of this network, Shimizu et al. [37] demonstrated a TLR4-dependent relationship between GPX4 suppression and ferroptosis-related lipid peroxidation in sepsis, whereas Kang et al. [32] inked GPX4-controlled lipid peroxidation with inflammatory cell death and cytokine release in polymicrobial sepsis. Thus, the network findings provide systems-level support for ferroptosis–inflammation crosstalk but do not establish direct modulation of these pathways by Fer-1.

Several limitations should be considered. Ferroptosis was evaluated indirectly through ACSL4 and GPX4 immunoreactivity and associated oxidative-stress changes. Direct measures of ferroptotic cell death, including tissue iron accumulation, lipid ROS or oxidized phospholipids, GPX4 enzymatic activity, and characteristic ultrastructural alterations, were not assessed. Accordingly, the present findings support ferroptosis-related alterations rather than definitive ferroptotic cell death. Moreover, because Fer-1 functions as a radical-trapping antioxidant, its protective effects cannot be attributed specifically or exclusively to ferroptosis inhibition. The study evaluated a single Fer-1 dose, only two treatment time points with seven animals per treatment group, and a short 6 h experimental period, limiting conclusions regarding treatment timing, long-term tissue protection, and survival. Intestinal permeability and tight-junction proteins were not evaluated, and the unexpected treatment-timing effect requires confirmation using additional administration intervals and serial sampling. Furthermore, the molecular docking and pathway analyses remain exploratory and require experimental validation.

This caution is particularly relevant given the findings of Van Coillie et al. [38], who reported that pharmacological inhibition of lipid peroxidation was protective in experimental non-septic multiorgan dysfunction but did not confer comparable protection in sepsis-induced multiorgan dysfunction. Nevertheless, the convergence of the biochemical, histopathological, and immunohistochemical findings, together with directly comparable evidence from Hou et al. [29], supports further investigation of ferroptosis-related lipid peroxidation and Fer-1 in sepsis-associated intestinal injury.

## 5. Conclusions

Fer-1 attenuated CLP-induced colonic injury and was associated with favorable changes in systemic oxidative and inflammatory markers, colonic histopathology, and ferroptosis-related ACSL4/GPX4 immunoreactivity. These findings are consistent with the involvement of ferroptosis-associated pathways but do not establish ferroptotic cell death or a ferroptosis-specific mechanism of Fer-1. The exploratory treatment-timing findings, which were limited to selected oxidative and inflammatory outcomes after adjustment for multiple comparisons, do not establish superiority of delayed administration or define an optimal therapeutic window. Further studies incorporating additional administration schedules and direct measures of ferroptosis are warranted.

## Figures and Tables

**Table 1:** Serum biochemical parameters in the experimental groups.

Parameter	Sham	CLP	CLP + DMSO	Fer-1 (30 min)	Fer-1 (2 h)	*p*	ε^2^
CRP (mg/L)	0.80 (0.30–1.70) ^a^	49.10 (35.60–69.80) ^c^	46.30 (36.70–66.70) ^c^	38.80 (34.50–55.40) ^bc^	24.30 (14.70–37.10) ^ab^	<0.001	0.785
LDH (U/L)	152.50 (127.10–312.50) ^ᵃ^	819.20 (721.30–1114.00) ^ᵇ^	875.10 (701.10–1649.30) ᵇ	418.10 (230.10–544.10) ^ᵃᵇ^	247.30 (189.20–351.40) ^ᵃ^	<0.001	0.827
TAS (mmol Trolox equiv./L)	0.61 (0.21–1.44) ^ab^	0.48 (0.17–0.53) ^a^	0.49 (0.37–0.71) ^ab^	0.71 (0.54–0.88) ^ab^	0.82 (0.45–0.93) ^b^	0.004	0.452
TOS (µmol H_2_O_2_ equiv./L)	5.30 (1.18–9.08) ^a^	33.45 (25.18–39.17) ^b^	30.45 (14.96–41.20) ^b^	21.10 (12.96–36.16) ^ab^	19.14 (10.17–28.50) ^ab^	<0.001	0.724
OSI	0.98 (0.08–2.52) ^a^	8.02 (4.75–19.68) ^c^	6.43 (2.11–9.79) ^bc^	2.54 (1.83–4.11) ^ab^	2.26 (1.20–3.85) ^a^	<0.001	0.742
MDA (nmol/mL)	3.96 (1.18–4.25) ^a^	7.49 (6.82–8.99) ^c^	7.92 (6.12–8.25) ^c^	7.12 (6.40–8.01) ^bc^	5.96 (5.03–6.56) ^ab^	<0.001	0.756
GSH (mg/L)	500.90 (379.80–589.41) ^c^	278.17 (150.09–294.59) ^a^	256.30 (201.18–287.42) ^a^	300.28 (243.80–393.80) ^ab^	394.59 (296.35–456.28) ^bc^	<0.001	0.757
SOD1 (ng/mL)	508.46 (369.84–588.34) ^c^	189.16 (144.18–302.16) ^a^	189.13 (96.18–287.18) ^a^	255.46 (149.16–311.69) ^ab^	366.32 (299.12–411.19) ^bc^	<0.001	0.737
CAT (ng/mL)	246.56 (201.27–289.15) ^c^	165.27 (113.12–196.14) ^a^	168.36 (131.49–201.12) ^ab^	189.27 (178.53–196.25) ^ab^	216.15 (201.01–254.79) ^bc^	<0.001	0.787

Values are presented as median (minimum–maximum). Comparisons were performed using the Kruskal–Wallis test followed by Dunn–Bonferroni post hoc analysis. Groups sharing at least one superscript letter are not significantly different (*p* > 0.05). ε^2^: epsilon-squared effect size.

**Table 2:** Serum inflammatory cytokine levels in the experimental groups.

Parameter	Sham	CLP	CLP+DMSO	Fer-1 (30 min)	Fer-1 (2 h)	*p*	ε^2^
TNF-α (pg/mL)	77.14 (56.18–89.14) ^a^	227.72 (147.56–318.14) ^c^	228.17 (176.48–289.00) ^c^	189.18 (147.56–208.78) ^bc^	114.25 (96.44–149.45) ^ab^	<0.001	0.821
IL-6 (pg/mL)	36.19 (28.12–42.56) ^a^	149.02 (112.28–156.47) ^c^	136.69 (108.27–152.89) ^bc^	103.24 (96.24–121.01) ^ab^	88.25 (76.24–96.78) ^a^	<0.001	0.874

Values are presented as median (minimum–maximum). Comparisons were performed using the Kruskal–Wallis test followed by Dunn–Bonferroni post hoc analysis. Groups sharing at least one superscript letter are not significantly different (*p* > 0.05). ε^2^: epsilon-squared effect size.

**Table 3:** Histopathological and immunohistochemical findings in the experimental groups.

Parameter	Sham	CLP	CLP+DMSO	Fer-1 (30 min)	Fer-1 (2 h)	*p*	ε^2^
Histopathological score	1.00 (0.00–1.00) ^a^	4.00 (4.00–5.00) ^c^	4.00 (3.00–5.00) ^bc^	3.00 (2.00–4.00) ^ab^	2.00 (1.00–3.00) ^a^	<0.001	0.848
ACSL4 H-score	63.80 (58.40–85.00) ^a^	211.40 (145.80–233.70) ^c^	192.40 (150.20–224.50) ^bc^	121.90 (103.20–155.60) ^ab^	108.03 (95.60–134.20) ^a^	<0.001	0.861
GPX4 H-score	212.20 (185.60–234.50) ^c^	98.90 (75.80–121.50) ^a^	113.40 (96.40–134.50) ^ab^	137.90 (112.50–147.50) ^ab^	152.60 (133.40–167.50) ^b^	<0.001	0.852

Values are presented as median (minimum–maximum). Comparisons were performed using the Kruskal–Wallis test followed by Dunn–Bonferroni post hoc analysis. Groups sharing at least one superscript letter are not significantly different (*p* > 0.05). ε^2^: epsilon-squared effect size.

**Table 4:** Group-adjusted partial correlations of ACSL4, GPX4, and histopathological scores with biochemical and inflammatory parameters.

Parameter	ACSL4 H-Score, r	*p*	GPX4 H-Score, r	*p*	Histopathological Score, r	*p*
CRP	−0.077	0.681	0.257	0.162	−0.133	0.477
LDH	−0.120	0.520	−0.101	0.587	0.205	0.269
TAS	−0.144	0.438	0.094	0.614	0.130	0.487
TOS	0.043	0.818	0.092	0.622	−0.155	0.406
OSI	0.286	0.119	0.083	0.657	−0.207	0.265
MDA	−0.128	0.491	0.022	0.906	0.294	0.109
GSH	0.007	0.972	−0.242	0.189	−0.010	0.957
SOD1	−0.176	0.345	0.020	0.915	−0.162	0.384
CAT	−0.073	0.696	−0.275	0.134	−0.030	0.872
TNF-α	−0.357	0.049	0.038	0.838	−0.129	0.489
IL-6	−0.382	0.034	−0.006	0.973	−0.037	0.842

Values are partial correlation coefficients adjusted for experimental group membership using four dummy-coded covariates, with the Sham group as the reference category (*n* = 35; df = 29). Two-sided *p* values are reported. These analyses were performed as sensitivity analyses to assess the influence of between-group experimental differences on the initial pooled correlations and should be interpreted as exploratory.

**Table 5:** Direct comparison of the Fer-1 treatment groups according to administration time.

Parameter	Fer-1 (30 min)	Fer-1 (2 h)	U	Unadjusted *p*	Holm-Adjusted *p*	Effect Size (*r*)
CRP (mg/L)	38.80 (34.50–55.40)	24.30 (14.70–37.10)	3.0	0.006	0.060	0.735
LDH (U/L)	418.10 (230.10–544.10)	247.30 (189.20–351.40)	6.0	0.018	0.144	0.632
TAS (mmol Trolox Eq/L)	0.71 (0.54–0.88)	0.82 (0.45–0.93)	15.0	0.224	0.568	0.325
TOS (μmol H_2_O_2_ Eq/L)	21.10 (12.96–36.16)	19.14 (10.17–28.50)	18.0	0.406	0.568	0.222
OSI (AU)	2.54 (1.83–4.11)	2.26 (1.20–3.85)	14.0	0.180	0.568	0.359
MDA (nmol/mL)	7.12 (6.40–8.01)	5.96 (5.03–6.56)	2.0	0.004	0.044	0.768
GSH (mg/L)	300.28 (243.80–393.80)	394.59 (296.35–456.28)	8.0	0.035	0.210	0.563
SOD1 (ng/mL)	255.46 (149.16–311.69)	366.32 (299.12–411.19)	3.0	0.006	0.060	0.734
CAT (ng/mL)	189.27 (178.53–196.25)	216.15 (201.01–254.79)	0.0	0.002	0.028	0.836
TNF-α (pg/mL)	189.18 (147.56–208.78)	114.25 (96.44–149.45)	1.0	0.003	0.039	0.803
IL-6 (pg/mL)	103.24 (96.24–121.01)	88.25 (76.24–96.78)	1.0	0.003	0.039	0.803
ACSL4 H-score	121.90 (103.20–155.60)	108.03 (95.60–134.20)	13.0	0.142	0.568	0.393
GPX4 H-score	137.90 (112.50–147.50)	152.60 (133.40–167.50)	7.5	0.030	0.210	0.581
Histopathological score	3.00 (2.00–4.00)	2.00 (1.00–3.00)	9.0	0.036	0.210	0.560

*p* values were obtained using two-sided Mann–Whitney U tests. To account for the 14 direct comparisons between the Fer-1 treatment groups, *p* values were adjusted using the Holm procedure. Statistical significance was determined based on Holm-adjusted *p* < 0.05.

## Data Availability

The data supporting the findings of this study are contained within the article.

## Supplementary Materials

The following supporting information can be downloaded at: https://www.mdpi.com/article/10.3390/cimb48090894/s1.
