# Supplementary material for: Ferrostatin-1 Attenuates CLP-Induced Colonic Injury: Associations with Ferroptosis-Related Alterations, Oxidative Stress, Inflammation, and Treatment Timing
Source: Curr Issues Mol Biol. 2026 Sep 1;48(9):894. doi: 10.3390/cimb48090894 (PMC13605974; doi:10.3390/cimb48090894)
Supplement: Supplementary file 1 [file cimb-48-00894-s001.zip › cimb-4477150-supplementary.pdf]

current issues in
molecular biology

MDPI

Article

Ferrostatin-1 Attenuates CLP-Induced Colonic Injury: Associations with Ferroptosis-Related Alterations, Oxidative Stress, Inflammation, and Treatment Timing

Mehmet Tolga Kafadar [1,*] and Eda Yıldızhan [2]

[1] Department of General Surgery, Faculty of Medicine, Dicle University, 21280 Diyarbakır, Turkey
[2] Department of Histology and Embryology, Faculty of Medicine, Recep Tayyip Erdogan University, 53100 Rize, Turkey; eda.yildizhan@erdogan.edu.tr
* Correspondence: drtolgakafadar@hotmail.com

Table S1. Summary of biochemical parameters, analytical methods, assay kits, and measurement units used in the study.

Parameter	Method	Manufacturer	Catalog No.	Unit
CRP	Biochemical assay	—	—	mg/L
LDH	Automated biochemistry	—	—	U/L
TAS	Colorimetric	Rel Assay	—	mmol Trolox Eq/L
TOS	Colorimetric	Rel Assay	—	μmol H_2O_2 Eq/L
MDA	ELISA	Bioassay Technology	E0156Ra	nmol/mL
GSH	ELISA	Bioassay Technology	EA0113Ra	mg/L
SOD1	ELISA	Bioassay Technology	E2269Ra	ng/mL
CAT	ELISA	Bioassay Technology	E0869Ra	ng/mL
TNF-α	ELISA	Bioassay Technology	E076Ra	pg/mL
IL-6	ELISA	Bioassay Technology	E0135Ra	pg/mL

Molecular analysis

Molecular docking analysis predicted that Fer-1 could be accommodated within a putative binding pocket of ACSL4 (Figure 4). The predicted docking pose positioned Fer-1 in proximity to several amino acid residues, including Glu158, Thr159, Ala182, Thr183, Tyr318, Pro320, Pro345, Leu346, Arg380, Thr679, Pro680, Glu681, Asp687, and Phe689. Visualization of the docked complex showed the ligand positioned within a defined cavity surrounded by these residues. This predicted pose was consistent with the docking score obtained using AutoDock Vina. However, the computationally predicted ligand orientation and residue proximity do not establish direct binding, binding stability, or functional modulation of ACSL4 and should be regarded as hypothesis-generating.

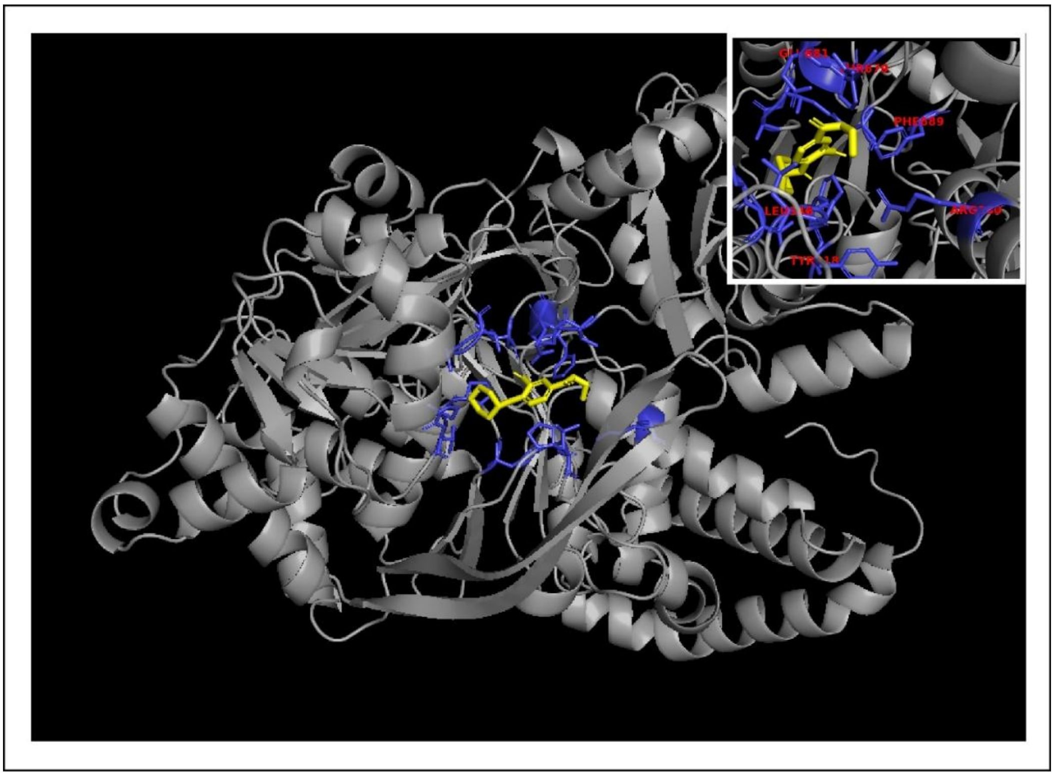

Figure S1. Molecular docking visualization of the predicted Ferrostatin-1–ACSL4 complex. The predicted three-dimensional structure of human ACSL4 is shown in cartoon representation (gray), whereas Ferrostatin-1 is displayed as yellow sticks. Inset: Amino acid residues located within 4 Å of the predicted ligand pose are shown as blue sticks and labeled to illustrate the putative binding

pocket. The docking pose shows the predicted positioning of Ferrostatin-1 within a cavity surrounded by the indicated residues.

Functional Enrichment Analysis

Functional enrichment analysis of the predefined protein set identified significant enrichment of pathways associated with innate immune activation and inflammatory signaling (Figure 6). The most significantly enriched KEGG pathways included the NF-κB signaling pathway, Toll-like receptor signaling pathway, NOD-like receptor signaling pathway, IL-17 signaling pathway, inflammatory bowel disease, and C-type lectin receptor signaling pathway. Cellular component analysis identified the NF-κB complex as the most significantly enriched compartment (FDR = 3.65×10^{-17}). These enrichment patterns provide functional context for the selected protein network; however, because the input proteins were predefined based on their known involvement in ferroptosis, oxidative stress, and inflammation, the findings should be interpreted as descriptive and hypothesis-generating rather than as independent evidence of Fer-1-mediated pathway modulation.

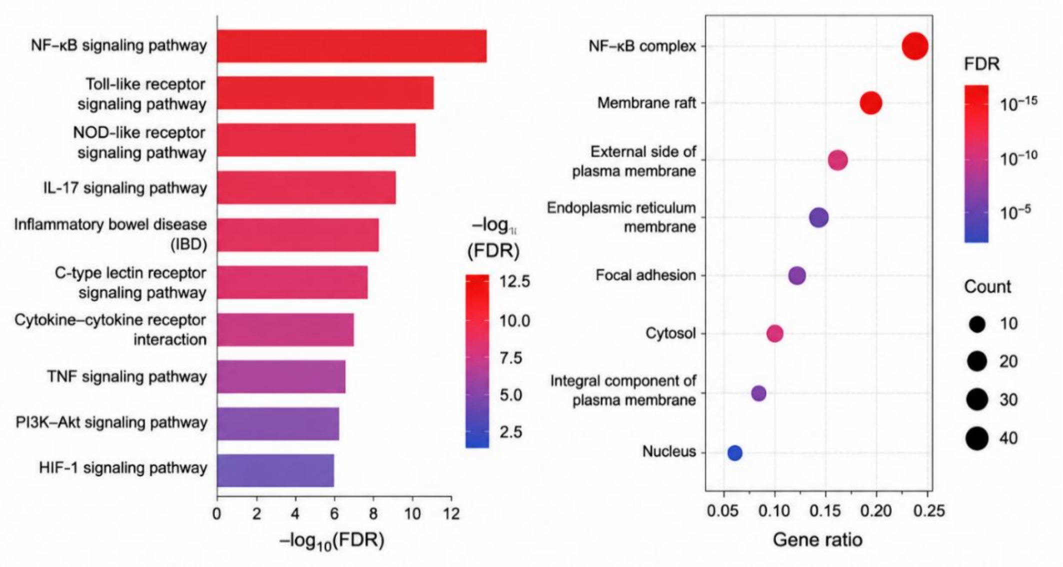

Figure S2. Functional enrichment analysis of the predefined ferroptosis- and inflammation-related protein network. KEGG pathway enrichment analysis showing the significantly enriched pathways (left); cellular component enrichment analysis showing the distribution of significantly enriched cellular compartments according to gene ratio (right).
